# Design and construction of a small embeddable nuclear magnetic resonance sensor utilizing 3D-printed components

**DOI:** 10.1016/j.ohx.2025.e00678

**Published:** 2025-07-13

**Authors:** Floriberto Díaz-Díaz, Prisciliano Felipe de Jesús Cano-Barrita

**Affiliations:** a CIIDIR Unidad Oaxaca, Instituto Politécnico Nacional, Calle Hornos No. 1003, Col. Noche Buena, Santa Cruz Xoxocotlán, Oaxaca 71230, Mexico; b Secretaría de Ciencia, Humanidades, Tecnología e Innovación (SECIHTI), Mexico

**Keywords:** ^1^H NMR sensor, 3D printing, Low-cost, Open source, Low-field

## Abstract

This paper presents the design and construction of a cost-effective embeddable nuclear magnetic resonance sensor using 3D printing to improve the construction process. The sensor comprises two 25.4 mm diameter x 3 mm thick neodymium-iron-boron disk magnets and an elliptical radio frequency coil. Magnetic field simulations were employed to determine the optimal separation between magnets, achieving a relatively homogeneous B_0_ field of 180 mT at the center of the array. Custom 3D-printed parts ensured precise magnet alignment and facilitated coil fabrication. The sensor was encased within a Faraday cage constructed from a printed circuit board to mitigate external electromagnetic interference. A remote tuning circuit was developed to tune the coil to 7.66 MHz. Initial testing involved using an eraser sample to determine the required 90° and 180° pulse amplitudes and duration. The sensor’s performance was further validated under immersion conditions in milk, yogurt, and fresh cement paste, using the Carr-Purcell-Meiboom-Gill technique. The signals obtained were processed by fitting the data to an exponential decay function to obtain the T_2_ lifetimes and their corresponding signal intensities, and by Inverse Laplace Transformation to obtain the T_2_ lifetime distribution. Results indicate the sensoŕs capability to detect variations in samples having different compositions.

## Graphical abstract

**Figure:**
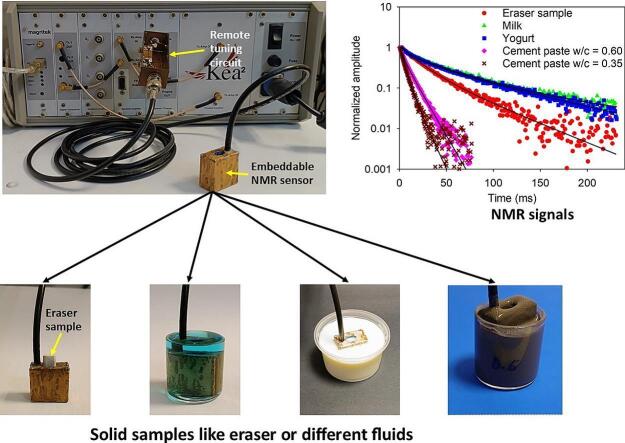

**Specifications table**

**Table:** 

Hardware name	Embeddable miniature NMR sensor
Subject area	• Engineering and materials science
Hardware type	• Measuring physical properties and in-lab sensors
Closest commercial analog	NMR MOUSE, NMR MOLE.
Open source license	*CC BY 4.0.*
Cost of hardware	*US$*132.67
Source file repository	https://doi.org/10.17605/OSF.IO/KYX5Q

## 1 Hardware in context

Nuclear magnetic resonance (NMR) is a non-destructive analytical technique used for the characterization of various materials due to its ability to analyze molecular and structural properties [1]. NMR relaxometry specifically enables the determination of relaxation times, which provide information about the microstructure and molecular dynamics [2], aspects that are fundamental in various industrial applications such as biomedicine [[3], [4], [5]], materials [6,7], and food quality control [[8], [9], [10]]. However, conventional NMR systems are typically characterized by high costs and substantial size, which consequently restrict their deployment in non-specialized environments and field research settings. In response to these limitations, the demand for compact, low-cost, and portable NMR sensors has surged in recent decades. Portable sensors, such as the NMR MOUSE [[11], [12], [13]], the three-magnet array [14,15], and the barrel-type magnet array [16,17], represent viable alternatives, expanding the scope of NMR applications to facilitate direct in situ measurements without necessitating sample transfer to a laboratory setting [18]. However, many of these portable configurations continue to face challenges regarding sensitivity, resolution, and overall cost. A notable limitation remains their lack of submersibility, which restricts their capacity to perform depth-variable measurements in liquids, thereby restricting potential applications in various fields. Furthermore, some designs involve complex construction, particularly within the magnet array [19], resulting in increased manufacturing difficulties and costs.

This work aims to present a low-cost embeddable NMR sensor capable of performing accurate measurements across a variety of sample types, encompassing both solid and liquid specimens. It is noteworthy that, for solid materials, only small samples are to be utilized within the sensor. This design constitutes a significant advancement over a previous design by the authors [20], as the integration of 3D printing technology enhances the precision and speed of constructing both the magnet array and radio frequency (RF) coil [21,22]. This results in maintaining the magnet́s separation and coil positioning within the homogeneous region of ​​the B_0_ static magnetic field. Moreover, using 3D-printed components simplifies the assembly process since the 3D-printed part for the RF coil fits within the magnet array, ensuring efficient and safe assembly.

To validate the sensoŕs performance, various measurements were performed using the Carr-Purcell-Meiboom-Gill (CPMG) technique [23] on a selection of samples, including rubber, milk, and yogurt. These tests evaluated the sensor's ability to detect relaxation properties and discern differences among samples with different characteristics.

**Fig. 1:**
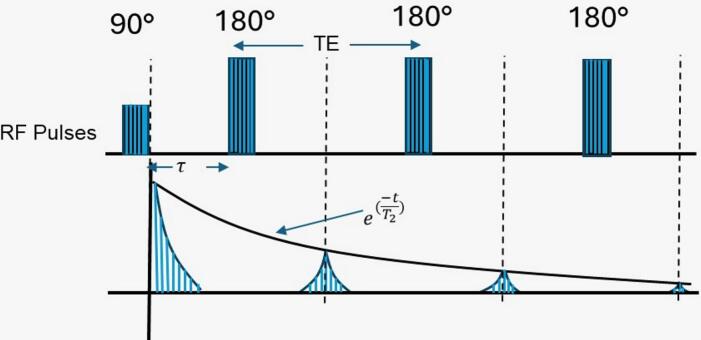
The CPMG sequence. The first two pulses are separated by a time τ, while the remaining pulses are separated by 2τ. Echoes occur halfway between the 180° pulses. TE is the echo time and is equal to 2τ.

The CPMG technique is used in NMR to measure the transverse magnetization decay (see Fig. 1). This sequence starts with a 90° pulse, followed by a series of 180° pulses applied at constant intervals. Each 180° pulse refocuses the magnetization, creating a series of echoes whose amplitudes decrease due to transverse relaxation processes. By analyzing this decay, we can estimate the T_2_ lifetimes and their corresponding signal intensities.

## 2 Hardware description

**Fig. 2:**
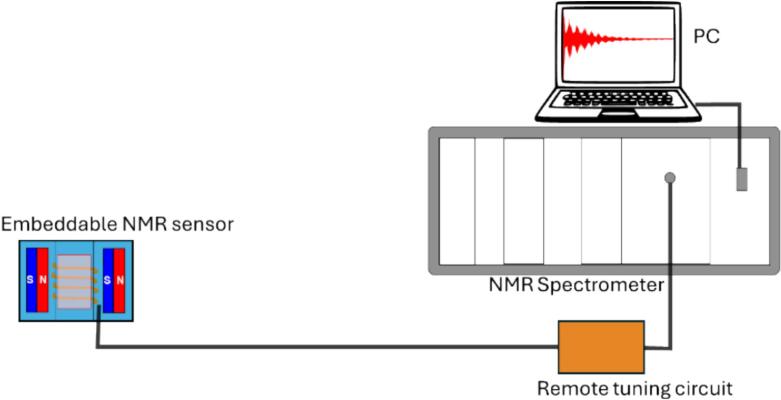
Schematic representation of the sensor remotely connected to the tuning circuit and the NMR spectrometer.

Fig. 2 shows a schematic overview of the embeddable NMR sensor. The system comprises a pair of magnets and a radio RF coil, all encased within the 3D-printed parts. The RF coil is connected to a remote tuning circuit (RTC) via an RG58 coaxial cable, which in turn is connected to the NMR spectrometer. The rationale for situating the tuning circuit away from the sensor lies in the potential for the sensor to be immersed in various samples. Such immersion can lead to variations in coil resonance frequency, primarily due to changes in impedance induced by the sample. By positioning the tuning circuit remotely, frequency adjustments can be performed without removing the sensor from the sample environment. Our embeddable NMR sensor can have the following applications:

• Measurement of NMR signals in liquid/colloidal samples such as fresh cement paste, polymer solutions, liquids, and gel-like materials, providing information on microstructure and molecular dynamics.
• In food quality control to evaluate the composition and quality of products such as milk and yogurt to monitor freshness, authenticity, and changes during storage.
• The sensor can also be used to study solid samples by introducing a small part of them into the sensor’s orifice.

## 3 Design files summary

**Table:** 

Design file name	File type	Open source license	Location of the file
*Magnet array 3D model*	*STL file*	*CC BY 4.0.*	https://doi.org/10.17605/OSF.IO/KYX5Q
*RF coil 3D model*	*STL file*	*CC BY 4.0.*	https://doi.org/10.17605/OSF.IO/KYX5Q
*PCB stencil*	*SVG and PDF file*	*CC BY 4.0.*	https://doi.org/10.17605/OSF.IO/KYX5Q

Design Files in the repository.

Magnet array 3D model is the.stl file to 3D print the part that secures the position of the magnets with a 10 mm separation.

RF coil 3D model is the.stl file to 3D print the part that comprises the RF coil structure.

PCB stencil files are the.SVG and.PDF files of the stencil necessary to cut the PCB pieces for the.

Faraday cage, and the PCB pieces for the remote tuning circuit.

## 4 Bill of materials summary

**Table:** 

Designator	Component	Number	Cost per unit −USD	Total cost −USD	Source of materials	Material type
*Tuning circuit*	*Nonmagnetic capacitor (33pF)*	1	4.490	4.490	Digikey	Ceramic
*Tuning circuit*	*Nonmagnetic capacitor (47pF)*	1	3.499	3.499	Digikey	Ceramic
*Tuning circuit*	*Variable capacitor (1-23pF)*	2	53.48	106.96	Digikey	Non-specific
*Tuning circuit*	BNC straight bulkhead 50 Ohm	1	1.93	1.93	Digikey	Non-specific
*Tuning circuit*	SMA connector 50 Ohm	1	1.92	1.92	Local hardware store	Non-specific
	BNC connector 50 OHM	1	3.51	3.51	Digikey	Non-specific
*Sensor*	Disc magnet	2	2.23	4.46	Local hardware store	NdFeB
	3D Printed Parts	1	2.95	2.95	Custom or local 3D printing service	Polymer (PLA)
	Printed circuit board (PCB) of 100 x 200 x 0.7 mm.	1	2.95	2.95	Local hardware store	Copper −Fiberglass
*Total*				132.67		

## 5 Build instructions

**Fig. 3:**
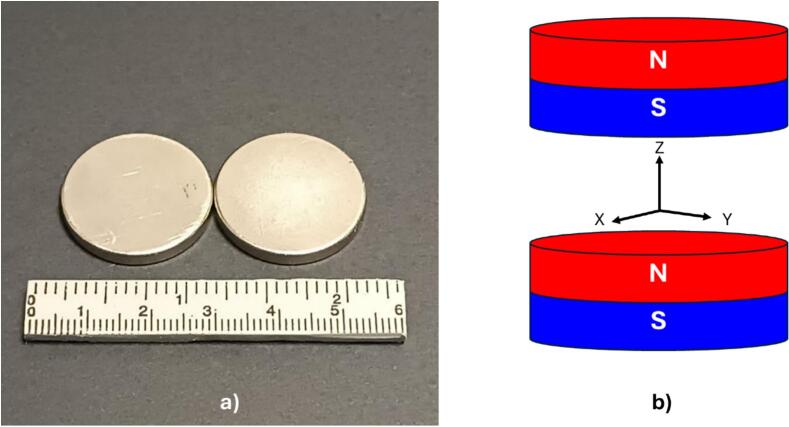
A) grade 35 neodymium-iron-boron (ndfeb) magnets used in the embeddable nmr sensor, b) magnets arrangement in the magnetic field simulation.

The first step of constructing the embeddable NMR sensor involved the selection and spatial arrangement of the magnets required for generating a B_0_ magnetic field. Specifically, two grade 35 neodymium-iron-boron (NdFeB) disc magnets were utilized, each with dimensions of 25.4 mm in diameter and 3.175 mm in thickness (Fig. 3a). To ascertain an optimal configuration for these magnets, magnetic field simulations were performed employing COMSOL Multiphysics® finite element analysis software (COMSOL, Burlington, MA, USA).

In the simulation setup, the geometry of the magnets array was defined by positioning the magnets in parallel with opposing poles (Fig. 3b). Subsequently, a surrounding volume, represented as a sphere with a diameter of 12.7 mm, was incorporated to model the air environment wherein the magnets would operate. Within the software, the material properties were specified, designating grade 35 NdFeB for the magnets and corresponding properties for the air medium within the sphere. The simulation involved adjusting the separation distance between the magnets to achieve optimal magnetic field homogeneity at the center of the array. Through systematic variation and analysis, it was determined that a separation distance of 10 mm between the magnets yielded the desired magnetic field homogeneity. It is noteworthy that this optimal separation may differ when using magnets of varying sizes or grades.

**Fig. 4:**
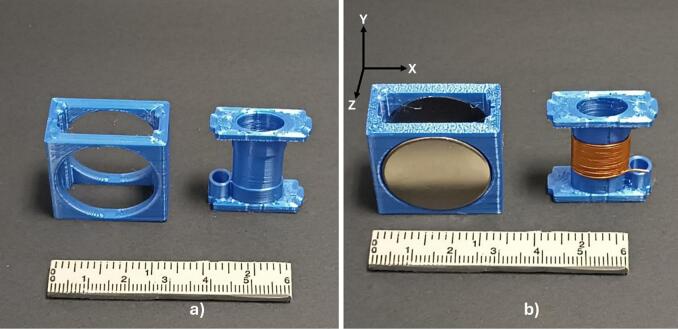
3D-prited parts and assembly of embeddable NMR sensor components. a) 3D-printed parts: The left 3D-printed part secures the position of the magnets with a 10 mm separation, and the right 3D-printed part forms the RF coil structure. b) The assembly process is depicted, illustrating the placement of the magnets within the first 3D-printed part alongside the elliptical solenoid coil situated in the second 3D-printed part.

After determining the spacing of the magnets, we used the free version of Autodesk Fusion © 2024 to create two 3D models, both of which were fabricated using 3D printing technology with polylactic acid (PLA) filament. The first 3D-printed part was designed to securely hold the magnets in specific positions established through magnetic field simulations, maintaining a separation of 10 mm between each magnet (Fig. 4a). The second 3D-printed part was created to aid in constructing the RF coil while providing the necessary structural support for its design (Fig. 4a). This 3D-printed part features an elliptical central tube designed winding the conductive wire, thereby forming the RF coil, alongside an aperture for the ingress of the coaxial cable intended for RF coil connectivity. Subsequent to the manufacturing process, the assembly of the magnet array was conducted using the first 3D-printed part, where the magnets were precisely positioned (Fig. 4b). After the magnet array was constructed, the magnetic field was mapped manually at the center of the array in the XY and ZY planes, using increments of 1 mm. A single-axis HT-20 gaussmeter (Hangzhou Best Magnet Co. Ltd, Zhejiang, China) was employed for this purpose. The elliptical solenoid coil, made with AWG 27 enameled copper wire, comprising a total of 19 turns, was built using the second 3D-printed part, as shown in Fig. 4b.

**Fig. 5:**
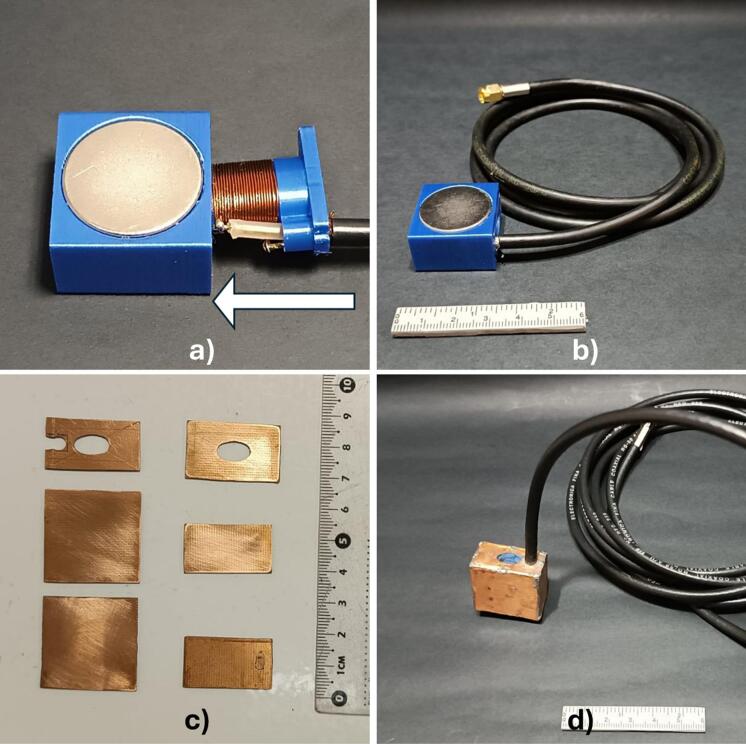
Sensor assembly process: a) the RF coil is moved in the indicated direction (white arrow); b) Assembled sensor; c) PCB pieces cut out to form a Faraday cage that shields the sensor from external RF interference; d) the sensor placed within the Faraday cage, with a visible aperture located at the top for the introduction of solid samples or liquids when the sensor is submerged.

Continuing with the sensor's construction, an adhesive (super glue) was used to securely attach the coil to the 3D-printed part. A coaxial connection cable (RG58, measuring 2.44 m in length) corresponding to a wavelength of λ/16 was subsequently soldered to the RF coil. In this case, the cable length is manageable and suitable for immersion experiments or direct sample placement within the sensor. In terms of efficiency, this length is short enough to reduce reflections, signal loss, and induced currents that could degrade signal quality. Following this, the RF coil was inserted into the magnet array. The design of the 3D-printed parts allows for a straightforward assembly process: the RF coil is simply slid into place to ensure a precise fit within the magnetic array, as demonstrated in Fig. 5a and 5b. This placement guarantees that the RF coil is centered within the homogeneous region of ​​the magnetic field. To create an electromagnetic interference shield similar to a Faraday cage and to cover the sensor, 0.7 mm thick printed circuit board (PCB) pieces were used. These pieces were cut according to the specifications outlined in the stencil available in the repository, as shown in Fig. 5c. These pieces were soldered with tin to ensure electrical continuity throughout the entire enclosure (Fig. 5d). Moreover, grounding of the board is achieved through the coaxial cable connection. To enhance the durability and waterproof the internal region housing the RF coil, a layer of epoxy resin was applied to the sensoŕs surface.

**Fig. 6:**
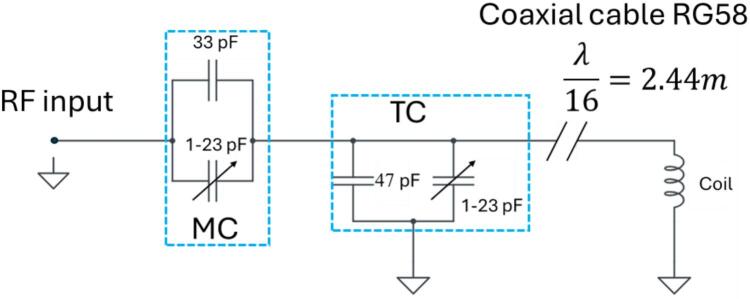
Electrical diagram of the RTC.

Due to its dielectric and conductive load, the RF coil's resonance frequency may be affected when the sensor is used in applications requiring immersion in a liquid sample. Furthermore, variations in temperature can affect the intensity of the magnetic field B_0_, resulting in fluctuations in the Larmor frequency. A remote tuning circuit (RTC), whose electrical diagram is shown in Fig. 6, was designed to tackle these challenges. This circuit enables frequency adjustment even when the sensor is submerged in the sample.

**Fig. 7:**
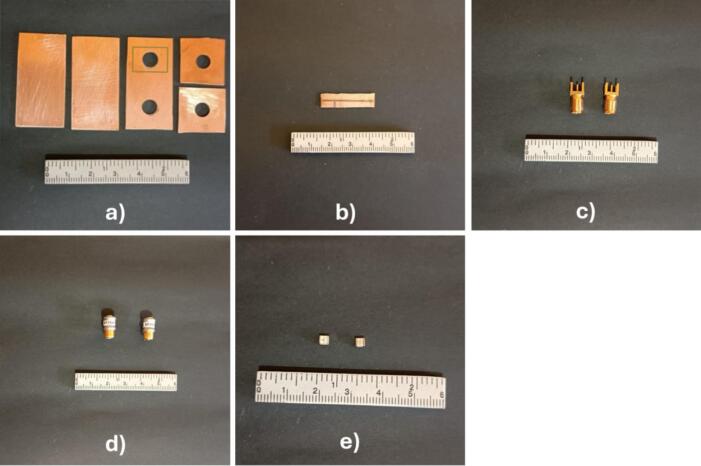
Materials used for the manufacture of the tuning circuit: a) PCB for the Faraday cage, b) PCB to solder the fixed capacitors, c) Female SMA connectors for the chassis, d) variable capacitors, and e) SMD fixed capacitors.

The materials required to construct the RTC, as illustrated in Fig. 7, include PCB boards cut to the specifications stencil available in the repository, two subminiature version A (SMA) female connectors, two variable capacitors ranging from 1 to 23 pF, and two fixed surface-mount device (SMD) capacitors (33 pF and 47 pF).

**Fig. 8:**
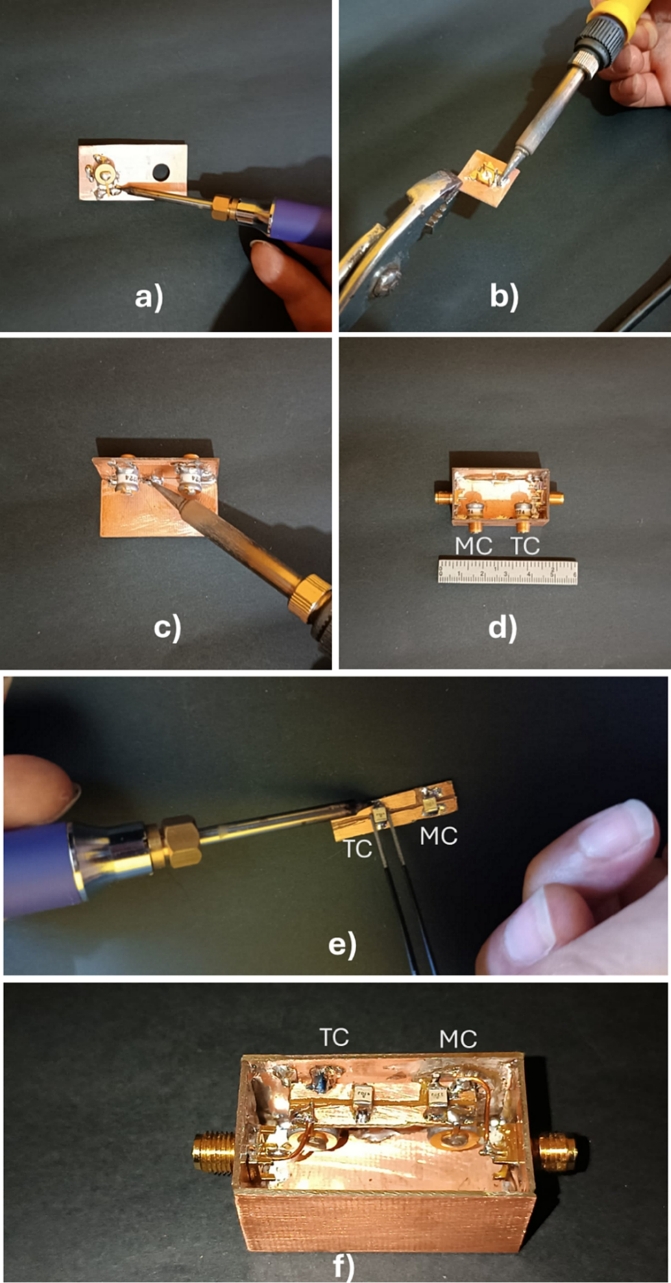
RTC assembly process, TC and MC indicate the position of tuning capacitors and matching capacitors respectively. a) Soldering the variable capacitors, b) soldering the SMA connectors, c) assembling the PCB boards to form the Faraday cage, d) the assembled Faraday cage with variable capacitors, e) soldering fixed capacitors, and f) Interconnection of all components.

The assembly process for the RTC is illustrated in Fig. 8. First, components such as variable capacitors and SMA connectors were soldered onto their respective boards (see Fig. 8a and Fig. 8b), which facilitated the overall assembly. Next, the Faraday cage was constructed by soldering the boards together (Fig. 8c). Once the cage was completed (Fig. 8d), fixed capacitors were soldered onto the corresponding board (Fig. 8e), which was then placed over the variable capacitors. Finally, the necessary connections were made to interconnect all the components (Fig. 8f), following the diagram shown in Fig. 6.

## 6 Operation instructions

**Fig. 9:**
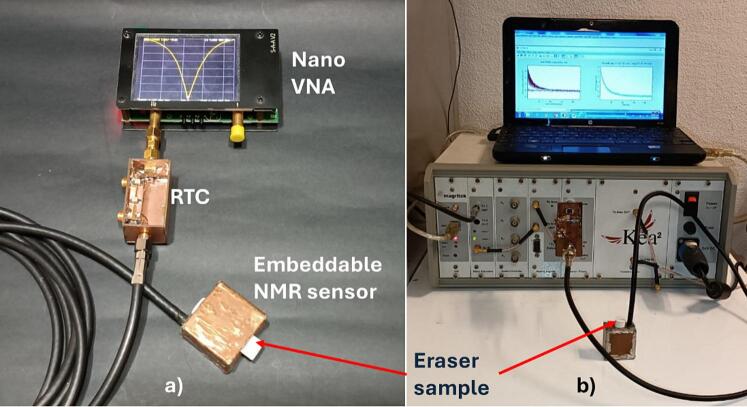
Sensor setup for use in a CPMG NMR experiment: a) RF coil tuning process using the RTC; b) sensor with RTC connected to an NMR spectrometer while running the CPMG experiment.

To utilize the sensor, the sample must be positioned within the designated cavity, or the sensor should be immersed within the liquid or a sample in a colloidal or fresh state. The sensor is subsequently connected to the remote tuning circuit, which in turn is connected to a vector network analyzer (Nano VNA V2_2, V2 Plus), as shown in Fig. 9a. It is important to note that the nanoVNA was used in this case because the developed sensor is designed for portable NMR spectrometers, which sometimes lack of a built-in RF coil tuning system. The nanoVNA is especially useful in these situations due to its affordability and accessibility. The RF coil is then tuned to the operational frequency of 7.66 MHz using the variable tuning and matching capacitors, thereby allowing the resonance frequency to be tuned and the impedance coupling to be adjusted. The sensor, in conjunction with the tuning circuit, is then connected to an NMR spectrometer; in our case, a Kea^2^ portable spectrometer was used (Magritek Limited, Wellington, New Zealand), shown in Fig. 9b, equipped with a 100 W RF amplifier. Subsequently, the necessary parameters for the CPMG technique are then set, which include amplitude, 90° and 180° pulse widths, operational frequency, number of scans, echo time, number of echoes, and repetition time. Once these parameters are set, the measurement process is initiated.

## 7 Validation and characterization

**Fig. 10:**
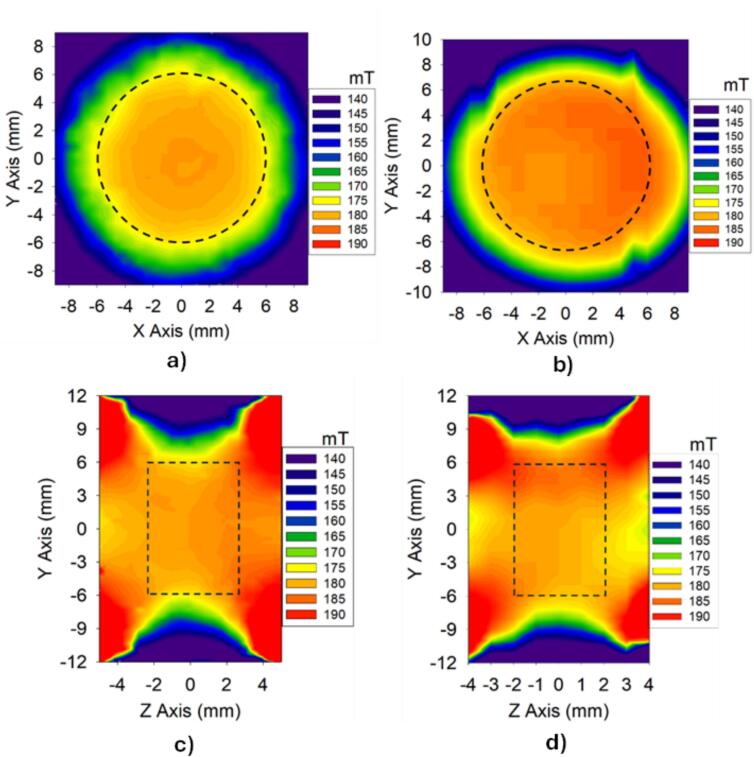
Characterization of the magnetic field generated by the magnet array of the embeddable sensor: a) Simulated magnetic field in the XY plane, b) Experimental magnetic field in the XY plane, c) Simulated magnetic field in the ZY plane, and d) Experimental magnetic field in the ZY plane. The dashed lines indicate the homogeneous magnetic field zone.

A comparison between the static magnetic field generated by the magnet array, encompassing both simulation results and experimental measurements, is illustrated for the XY (Fig. 10a) and XZ (Fig. 10b) planes. It is observed that in both representations, there is a cylindrical volume where the magnetic field is relatively homogeneous, with an approximate magnitude of 180 mT. This volume was considered when designing the geometry of the RF coil. The dimensions of this homogeneous space are approximately 12 mm in diameter and 5 mm thick. The evaluation of the magnetic field homogeneity in the region of interest yielded values of 14,656 ppm from the numerical simulation and 15,734 ppm from the experimental measurements, which are relatively close. This homogeneity places the developed sensor at a competitive level compared to commercial systems such as the NMR-MOLE [17], which has a homogeneity of 15,000 ppm. Therefore, the sensor developed in this research is competitive and offers adequate performance for these types of measurements.

**Fig. 11:**
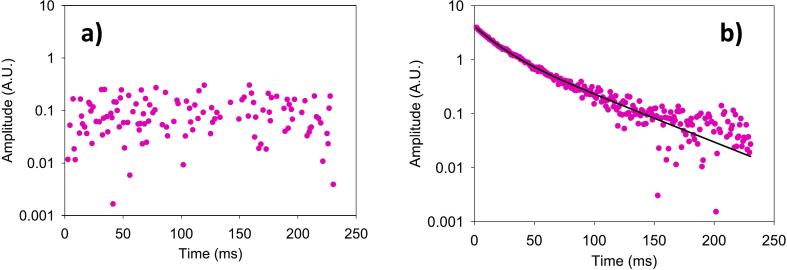
Signals obtained with the CPMG technique a) without sample and b) with an eraser sample.

Measurements using the CPMG technique were performed on different materials to assess the performance of the sensor. Prior to characterizing different materials, an eraser sample was chosen to determine the amplitude and pulse width values ​​(pulse width = 9 μs, 90° pulse amplitude = 7.94 W, and 180° pulse amplitude = 31.62 W) since this material does not undergo significant changes in consistency during the test time and contains abundant proton in its chemical structure. The validation involved measurements both with and without sample present in the sensor (Fig. 11), using the parameters shown in Table 1. In the absence of a sample, the output signal was noise (Fig. 11a). In contrast, the introduction of the sample resulted in the detection of an exponential decay signal characteristic of the transverse relaxation of the magnetization vector (Fig. 11b).

**Table 1:** Parameters used to measure samples using the CPMG technique.

	Eraser	Milk	Yogurt	Cement paste
Pulse width (μs)	9	9	9	9
Number of echoes	256	512	512	256
Echo time (μs)	900	900	900	300
Number of scans	512	128	128	512
Acquisition time (min)	5.55	2	2	4.28

**Fig. 12:**
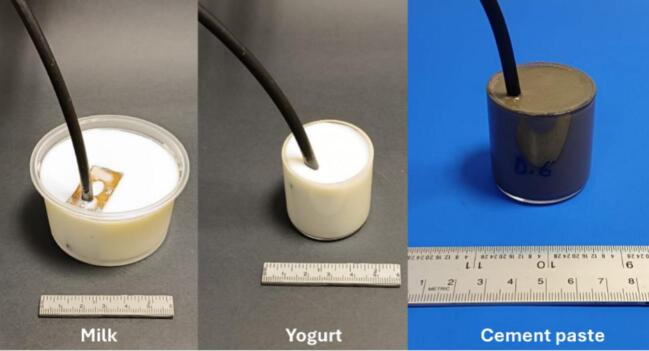
Sensor immersed in milk, yogurt and fresh cement paste during CPMG NMR measurements.

To evaluate the sensor's performance under immersion conditions, tests were performed on milk, yogurt, and fresh cement paste samples (Fig. 12). The cement paste was prepared with water-to-cement (w/c) ratios of 0.60 and 0.35. The fluid consistency of these samples enabled immersion of the sensor for measurement using the CPMG technique. It is important to note that the milk and yogurt used in these experiments were purchased separately as commercial products. The parameters employed for the measurements in these samples are shown in Table. 1. The signals were fitted to multi-exponential decay functions according to Equation (1), obtaining the T_2_ lifetime components ​​and their corresponding signal contributions, as shown in Table 2.

(1) $$S\left(t\right)=\sum_{i}M_{0,i}e^{-t/T_{2,i}}$$

where S is the signal intensity, t is time, M_0,i_ is the initial magnetization of the i-th component and T_2,i_ is the T_2_ lifetime of the i-th component.

**Table 2:** T_2_ values ​​and percentage contribution of the components obtained for each sample measured.

Sample	T_2,1_(ms)	T_2,2_(ms)	T_2,3_(ms)	T_2,4_(ms)	M_0,1_(%)	M_0,2_(%)	M_0,3_(%)	M_0,4_(%)
Eraser	3.6	18.21	46.29	−	12.82	58.85	28.31	−
Milk	1.54	11.32	48.54	169.49	17.43	24.6	48.49	9.46
Yogurt	0.75	7.98	25.83	81.3	37.62	11.17	26.22	24.97
Cement paste w/c = 0.60	1.14	5.19	10.9	−	13.66	25.76	60.57	−
Cement paste w/c = 0.35	0.76	4.17	8.79	−	12.23	62.56	25.2	−

The T_2_ lifetimes measured for each sample agree with expectations based on the mobility of the protons and chemical composition of the studied materials. Samples with high molecular mobility, such as milk and yogurt, exhibited longer relaxation times (T_2_ > 50 ms), primarily due to the presence of less constrained protons in water, fat, proteins, etc. In contrast, samples with low molecular mobility, like the eraser and the cement paste, displayed significantly shorter relaxation times (T_2_ < 10 ms). For the fresh cement paste, these short times can be attributed to the presence of iron in ordinary Portland cement that reduces the T_2_ lifetimes, and to the interaction between water molecules and the hydrating cement particles.

**Fig. 13:**
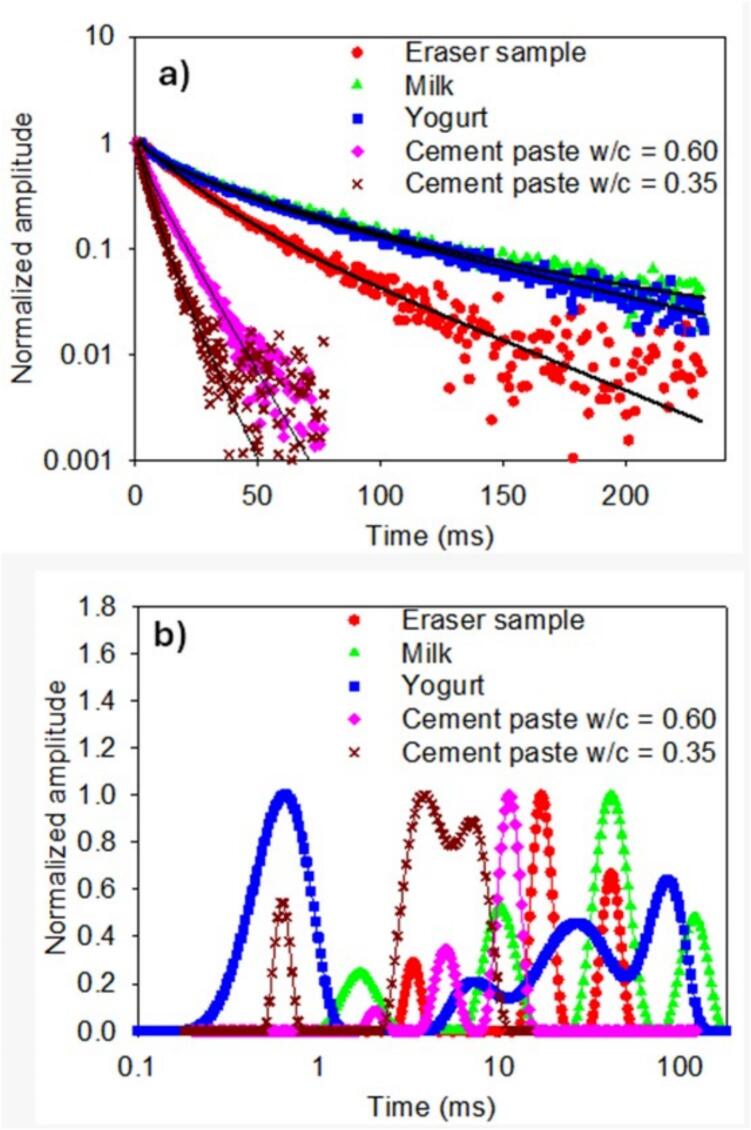
A) cpmg nmr signals measured and b) inverse laplace transform of the signals in a).

Fig. 13a shows all the obtained signals, which were analyzed using the inverse Laplace transform [24]. This analysis revealed four distinct components within the T_2_ distribution for milk and yogurt, three components for both the cement paste and for the eraser sample (Fig. 13b). The T_2_ lifetimes in Table 2 obtained by fitting the CPMG decays to multiexponential decay functions, serve as basis to compare with the T_2_ lifetime distributions obtained with the Inverse Laplace Transform (ILT), resulting in a close agreement between the mean T_2_ value of each population in the ILT data with the T_2_ values from the fitting to exponential decay functions. These results underscore the sensoŕs capability to differentiate the relaxation properties of each sample based on their unique compositional characteristics. The T_2_ differences obtained between milk and yogurt, and between cement paste with different w/c ratios, indicate that the sensor can detect proton interactions in different molecular environments. Therefore, results indicate promising application performance for the characterization of samples that may exhibit microstructural/chemical changes over time.

## CRediT authorship contribution statement

**Floriberto Díaz-Díaz:** Writing – original draft, Validation, Software, Methodology, Investigation, Conceptualization. **Prisciliano Felipe de Jesús Cano-Barrita:** Writing – review & editing, Visualization, Supervision, Data curation, Conceptualization.

## Declaration of competing interest

The authors declare that they have no known competing financial interests or personal relationships that could have appeared to influence the work reported in this paper.
